# A Practical Treatis One Dental Medicine

**Published:** 1853-03

**Authors:** 


*A Practical Treatise on Dental Medicine*, being a compendium of Medical Science, as connected with the study of Dental Surgery, to which is appended an inquiry into the use of Chloroform and other Anæsthetic agents, second edition, revised, corrected and enlarged. By THOS. E. BOND, A.M. M.D., Professor of Special Pathology and Therapeutics in the Baltimore College of Dental Surgery: Phil. Lindsay & Blackiston, 1852.

VERILY of making books there is no end. Every department of science and literature seems to be in labor; in the majority of cases the result being abortion, as is evidenced by imperfect development, and in many instances the entire inability of the little nursling to maintain life, though cared for tenderly by the whole family of motherly editors. On the other hand, we are pleased when we see produced by a healthy vigorous mind, a full grown, well-proportioned and perfectly developed offspring, which needs no puffing, no artificial respiration to make it live.

Dr. Bond's book is certainly not one of the first class. It is a work which must find favor with Students of Dentistry, and which, in the absence of anything more full and complete, cannot be dispensed with by those in the practice of that department of therapeutic science. We are glad to see a recognition of the fact, that mechanical skill alone is not sufficient, and that to be a good dentist, a man must understand the chemico-physiology and pathology of the structures with which he has to do, as well as the morbid conditions and curative indications of adjacent parts dependent on a diseased condition of the teeth themselves.

The book does not profess to contain every thing that can be said on the subject; indeed it is a pioneer, a first effort to embody facts and present principles, with special reference to dental pathology and therapeutics.

The edition before us contains a chapter on anæsthetics, which will enhance the value of the work. In reference to that subject he says:

"I am convinced that *anæsthetics should always be administered to the patient in the recumbent position.*

They should *never be administered to a patient in whom the heart's action is at the time very feeble, whether from dread or otherwise.*

*They should never be administered to a patient subject to fainting fits.*"

Dr. Bond thinks that one common cause of death is syncope, produced by the administration of the Anæsthetic in an erect position, and from the fact that parturient females almost always inhale the anæsthesia in a recumbent position; he explains their singular exemption from the perils which have attended the use of these agents in other patients. J.

